# PMSNet: Multiscale Partial-Discharge Signal Feature Recognition Model via a Spatial Interaction Attention Mechanism

**DOI:** 10.3390/s24113342

**Published:** 2024-05-23

**Authors:** Yi Deng, Jiazheng Liu, Kuihu Zhu, Quan Xie, Hai Liu

**Affiliations:** 1 School of Electronic and Electrical Engineering, Wuhan Textile University, Wuhan 430200, China; 2 State Key Laboratory of New Textile Materials and Advanced Processing Technologies, Wuhan Textile University, Wuhan 430200, China; 3 Faculty of Artificial Intelligence in Education, Central China Normal University, Wuhan 430079, China

**Keywords:** partial discharge, multiscale, PRPD, extracted features, feature fusion

## Abstract

Partial discharge (PD) is a localized discharge phenomenon in the insulator of electrical equipment resulting from the electric field strength exceeding the local dielectric breakdown electric field. Partial-discharge signal identification is an important means of assessing the insulation status of electrical equipment and critical to the safe operation of electrical equipment. The identification effect of traditional methods is not ideal because the PD signal collected is subject to strong noise interference. To overcome noise interference, quickly and accurately identify PD signals, and eliminate potential safety hazards, this study proposes a PD signal identification method based on multiscale feature fusion. The method improves identification efficiency through the multiscale feature fusion and feature aggregation of phase-resolved partial-discharge (PRPD) diagrams by using PMSNet. The whole network consists of three parts: a CNN backbone composed of a multiscale feature fusion pyramid, a down-sampling feature enhancement (DSFB) module for each layer of the pyramid to acquire features from different layers, a Transformer encoder module dominated by a spatial interaction–attention mechanism to enhance subspace feature interactions, a final categorized feature recognition method for the PRPD maps and a final classification feature generation module (F-Collect). PMSNet improves recognition accuracy by 10% compared with traditional high-frequency current detection methods and current pulse detection methods. On the PRPD dataset, the validation accuracy of PMSNet is above 80%, the validation loss is about 0.3%, and the training accuracy exceeds 85%. Experimental results show that the use of PMSNet can greatly improve the recognition accuracy and robustness of PD signals and has good practicality and application prospects.

## 1. Introduction

Safe and reliable power equipment provides security for people’s lives, especially in densely populated cities with extremely high power loads. High-voltage power equipment, such as transformers and reactors, plays a vital role in regional transmission networks, and we need to ensure that they can operate stably and safely for long periods [1]. Insulation defects and degradation can seriously affect the performance and lifetime of these equipment. Partial-discharge signals that lead to insulation failure have become a diagnostic indicator for assessing the health of equipment systems. Partial discharges pose a serious threat to the development of fields such as the aerospace power chain where certain power systems located in specific hazardous areas may suffer from partial discharges that can lead to fires [2]. In forested areas, the lack of maintenance of basic power distribution facilities, coupled with long-term exposure to the elements, can result in partial discharges (PDs), thus posing a potential fire hazard [3].

PD is an electrical phenomenon originating from localized arc discharges within insulating materials. These discharges represent the predominant factor behind insulation failures observed in power transformers, cables, and other critical power equipment. They manifest as minute electrical discharges occurring either on the surface or within the insulating material itself, particularly under conditions of elevated voltages. Such tiny discharges can erode the insulation structure, leading to a localized increase in the temperature of the power equipment insulation and causing fires. Partial discharges in power equipment, if not resolved or detected in time, will continue to expand the deterioration area and eventually lead to overall insulation breakdown [4,5,6,7]. Partial discharges pose a serious threat to the safe operation of power equipment, and the ability to accurately detect localized discharge signals promptly is a critical step. Early detection of the type of PD signal is conducive to the targeted repair and maintenance of power equipment, effectively prevents further deterioration of equipment, and reduces the risk of accidents. A partial discharge is a complex physical process. In addition to the transfer of charge and the loss of electric energy, it also produces electromagnetic radiation, ultrasonic waves, light, heat, and new products [8]. An electrical analysis showed that when the discharge is generated, there is charge exchange, electromagnetic wave radiation, and energy loss at the discharge. The most obvious are reflected at both ends of the sample; when voltage was applied, there was a weak pulse voltage [9]. 

**Figure 1:**
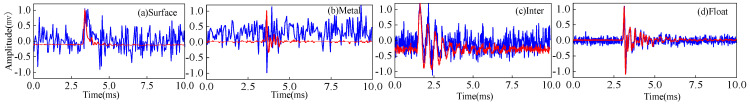
Partial-discharge signal: (**a**) surface PD signal; (**b**) metal PD signal; (**c**) inter-PD signal; and (**d**) float PD signal.

PD signals acquired by sensors carry noise and interference that can cause difficulties in signal identification When we applied our previously proposed adaptive variational mode decomposition (AVMD) method [10] on PD signals detected by infrared sensors, we found that the PD signals acquired by sensors carry noise and interference that can cause difficulties in signal identification. The discharge types are shown in Figure 1, where the blue curve is the original localized discharge signal with noise that we acquired through the sensor, and the red curve is the signal after we filtered and denoised it by using the AVMD algorithm. Considering that denoising and reconstructing the signal for each recognition greatly increases the workload, we transformed the 1D LD signal into a 2D phase-resolved partial-discharge(PRPD) graph.

**Figure 2:**
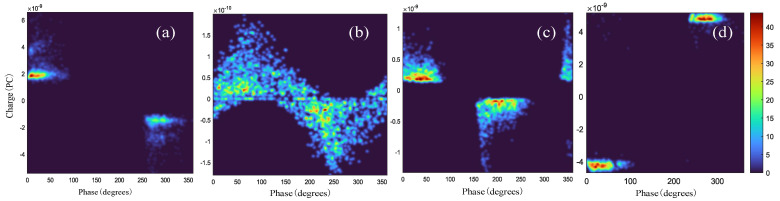
Phase diagram of localized discharges: (**a**) surface PRPD signal; (**b**) metal PRPD signal; (**c**) inter-PRPD signal; and (**d**) float PRPD signal.

As shown in Figure 2, noise predominantly affects the distribution of discharge phases and the frequency of discharges. Higher brightness indicates denser discharge occurrences. The representation of random noise in PRPD is not obvious. Due to its randomness, the color of its distribution is not bright. Recognition of the PRPD graph not only requires a consideration of the dependence relationship between local and global features but also requires learning the distribution of the different shades of colors in the PRPD graph, which greatly increases the workload. The features of fine edges in the PRPD graph are difficult to learn, and different fine-grained features must be captured by scale transformation, which is studied in this work.

The identification methods of localized signals can be traced back to the earliest physical methods of detecting signals and identifying the types of localized signals, among which the most typical is to use the pulse current method to detect localized signals and obtain the characteristics of the localized signals by analyzing various parameters of signal waveforms through the pulse sampling method of PD currents [11]. The current pulse sampling method can effectively identify some types of partial signals, but it is ineffective in the determination of noise. Although the current pulse sampling method can effectively identify some types of partial discharges, it is not effective in determining PD signals with high noise overlap. The effect of PD signals with high noise mixing is not ideal; therefore, in recent decades, the ultra-high-frequency (UHF) sampling method has gradually replaced the current pulse sampling method and has become the mainstream method. PD detection based on UHF sensors is more suitable for onsite detection than earlier traditional methods because it is free from external electromagnetic interference, which is beneficial for on-site insulation detection [12,13]. Moreover, the accuracy of the UHF method for detecting PD signals is higher than that of the pulse current method because it determines the signal type by processing UHF pulse amplitude sequences and time sequences to determine characteristic parameters [14]. The traditional partial-discharge signal identification method is relatively direct. It determines the characteristic parameters of the partial-discharge signal after detecting the PD waveform so as to determine the type of discharge. This detection method is direct and effective, but it lacks optimization, learning, and effect feedback, and the accuracy rate is average. Thus, scholars have developed pattern recognition of the partial-discharge signal on the basis of traditional partial-discharge signal identification. For example, the particle swarm optimization algorithm based on variable modal decomposition performs modal decomposition of the localized signal, calculates the sample entropy of each modal component, forms the localized signal features, and realizes localized signal detection through the particle swarm optimization parameter [15]. Ricardo et al. proposed a separation method of localized signals and extracted the features of the PRPD spectrum to perform identification measurements of localized signals [16]. Pattern recognition of localized signals has achieved remarkable results, and better results have been obtained through the automatic analysis of the signal data structure and features of the optimization iteration. The method is highly referential, direct, and effective, and its recognition accuracy is high. However, it ignores the 2D features of the PRPD map such as the size of the pixel value and the depth of the number of channels. This study proposes a form of PMSNet for the 2D-image features of localized signals. The proposed PMSNet analyzes the LD signal concerning the 2D-image features; it exploits deep feature information and improves recognition accuracy. In recent years, there has been a shift in the identification of localized discharge signals from mere sensor indicator detection to a more sophisticated analysis of signal parameters. However, certain challenges persist, including a limited number of judgment indicators, low accuracy rates, and difficulties in deployment.

With the continuous development of computer technology and the emergence of deep learning methods in recent years [17], partial-discharge signal recognition methods are being constantly updated and developed. Traditional PD recognition methods have poor accuracy in practical engineering applications, and some changes need to be made. Machine learning methods (e.g., neural networks, expert systems, and fuzzy systems) are effective for localized signal detection and recognition [18,19]. Deep learning methods with high recognition accuracy and robustness have been applied to partial-discharge signal recognition. For instance, deep learning techniques based on convolutional neural networks (CNN) and recurrent neural networks (RNN) have been successfully applied to the recognition of localized discharge signals, and commendable results have been obtained [20,21,22]. In partial-discharge signal recognition, traditional detection methods have limited parameter optimization ability and cannot further improve the recognition efficiency and accuracy. Zhang proposed a partial-discharge recognition method based on CNN that extracts the spatial features of partial-discharge signals through state–space reconstruction and improves the recognition efficiency of partial-discharge signals [23]. The CNN-based method incorporated with the long short-term memory (LSTM) network to recognize partial-discharge signals has also achieved success [24]; it uses CNN and the LSTM network to extract the spatial and temporal features of PD signals, respectively, and performs PD signal classification through the interaction of the two features. CNN extracts the PD signal features through layer-by-layer convolution, pooling, and other operations. In 2012, Li et al. proposed a UHF PD signal recognition method based on multiscale features [25]. This approach involves performing wavelet-transform-based multiscale decomposition of PD signal waveforms captured by UHF sensors, calculating the multiscale fractal dimensions and energy parameters of PD signals, and categorizing them accordingly. It exemplifies the application of the concept of multiscale analysis to PD signals. 

Multiscale feature extraction can extract features of different levels and suppress the detail feature blur caused by multi-layer network convolution. At present, multiscale feature fusion is an innovative direction in the field of artificial intelligence. In this study, we adopt the research idea of scale feature fusion and realize multiscale feature fusion for PRPD graphs through the backbone network of CNN plus Transformer in order to capture the dependency relationship between local signal features and improve recognition accuracy and efficiency.

The method used in this research is a joint network structure of CNN and Transformer for the multiscale feature extraction of PRPD graphs. The CNN part consists of a multiscale feature fusion pyramid. The bottom of the pyramid represents the shallow features, and the process of going from the bottom to the top of the pyramid represents the process of the features transitioning from shallow to deep. Multiscale feature fusion is performed by extracting the features at different levels in the network. In this study, Transformer is used to learn the features through a spatial interactive attention mechanism (SI-Attention) that focuses on the main features of the object, suppresses the useless features, and generates a categorized feature through the feature generation module (F-Collect) to implement the recognition task. The contributions of this work are as follows:

(i) The Vision Transformer neural network structure is constructed to realize multiscale feature extraction for PRPD signals and promote the combination of PD identification research and computer science;

(ii) The study utilizes a CNN-based feature fusion pyramid network to extract multiscale features from PRPD localized discharge signals. The SI-Attention mechanism is devised to enhance the interplay between these features. The F-Collect module generates classification features;

(iii) The proposed PMSNet obtained good results in the PRPD dataset test, with a recognition accuracy of 85.2% in the PRPD test set and a training loss of less than 0.3%, which is about 5% higher than the accuracy of similar PD recognition models.

The paper is structured as follows. Section 2 provides an overview of relevant studies on partial-discharge pattern recognition, Transformer, and attention mechanisms. Section 3 presents a detailed description of the proposed PMSNet. Section 4 introduces experiments that measure and compare the performance of the proposed PMSNet with that of state-of-the-art methods and discusses the challenges and weaknesses that require further research. Conclusions and future work directions are given in Section 5.

## 2. Related Work

### 2.1. Partial-Discharge Signal Identification

The recognition of partial-discharge signals is a multifaceted task that encompasses data preprocessing, feature extraction, pattern recognition, and other related procedures. Given that the acquired PD signal is full of noise and the waveform characteristics of the PD signal after denoising are not obvious, in this study, the PD 1D signal is converted into a 2D PRPD signal for preprocessing. Traditionally, PD signals are detected and recognized by amplitude thresholding, but different types of PD events may have different amplitudes and phase angle features, and the accurate extraction of these hidden and important features can greatly improve the recognition accuracy. Feature extraction and pattern recognition of PRPD signals have always been challenging. The backpropagation (BP) network is a widely employed neural network for the recognition of PD signals. The high-voltage features of PRPD map statistics based on the BP neural network were investigated using different sensor data. Sukma et al. varied the input patterns in different types of sensors and used the BP neural network for partial signal classification. A comprehensive study was conducted, and the experimental surface BP neural network was used for localized signal recognition with good results [26]. Herath et al. conducted a comparative study on the classification of PDs in generator insulation by using supervised maximum likelihood classifiers incorporated with statistical features derived from PRPD analysis [27]. Kunicki et al. proposed a method for selecting the defects of power transformers that uses acoustic emission signals (different types of PDs and typical defects) in a two-stage maximum likelihood classification method. Support vector machine (SVM; cubic, kernel, or cubic–kernel function), which utilizes energy features extracted by discrete wavelet transform as an input, had a better performance than k-nearest neighbor, decision tree (DT), and integrated bagging DT [28]. 

CNN is a prevalent deep learning model that is extensively employed in computer vision. It has excellent performance and numerous advantages in image processing and pattern recognition, including image recognition, target detection, and video recognition. Zhang et al. proposed a CNN-based partial-discharge recognition method that extracts the spatial characteristics of PD signals through state–space reconstruction, performs equivalent transformation of localized discharge signals per unit power period, and increases the introduction of the time domain, entropy, and geometric features to improve the recognition accuracy of localized discharge signals [20]. Li et al. proposed a multilinear CNN with a multiresolution UHF spectrum for PD signal classification [29]; it utilizes an LSTM network to fuse embedded multisensor information and mitigates the risk of overfitting. N. Puspitasar recognized experimentally obtained time-domain PD waveform images by means of an ordinary CNN structure, and the recognition accuracy exceeded 78% [30]. The CNN-based addition of the LSTM network to recognize PD signals was successful. CNN and the LSTM network are used to extract the spatial and temporal features of PD signals, respectively; classification of PD signals is realized through the interaction of the two features.

CNN remains one of the most important and effective models in computer vision. The multiscale feature fusion pyramid in this study is also based on the backbone network of CNN to introduce residual connectivity between levels, thus separating different levels of features from shallow to deep, mining the channel and pixel relationships of different scale features, and performing feature fusion to obtain multiscale fusion features [31].

### 2.2. Vision Transformer

In this study, the model of Transformer is utilized, but some improvements are made on the original model. Transformer is a neural network model based on the self-attention mechanism. Proposed by Vaswani et al. in 2017, it was initially used for natural language processing tasks. The self-attention layer in the Transformer model captures the relationships between different input sequences and the relationships between positions while performing parallel computing [32]. Vision Transformer’s structure draws on the design idea of Transformer and applies it to the image field, and it has achieved remarkable results [33]. After the proposal by VIT, researchers further improved and extended it. Li et al. proposed the T2T-VIT network structure. It captures global information by adding extra special tokens before the input path of VIT. The extra-special tokens represent the global content of the whole image and act as an aggregation point for all path information [34]. In this way, Transformer can model global and local features to effectively capture the details and overall information of the image. The CvT network architecture integrates CNN into Transformer to take full advantage of CNN’s convolutional layer and uses it to efficiently learn the hierarchical representation of the image [35], introduce convolution into Visual Transformer to efficiently capture the local information, and incorporate convolution to enhance the model’s sensitivity to fine-grained images. In this study, we utilize CNN and the improved Transformer structure for the feature extraction of PRPD maps. This work explores the use of a hybrid structure of CNN and Transformer to fully utilize their respective strengths and achieve the desired accuracy rate.

The method of exploring long-distance dependencies between features through Transformer is rarely used in partial-discharge recognition tasks, but in this study, high accuracy is obtained for feature learning and the extraction of PRPD maps by using CNN and the improved Transformer structure. The SI-Attention attention mechanism, introduced in the Transformer structure of this paper, can effectively segment the input feature vector according to the number of attention heads, redistribute the feature weight of each subspace, and establish cross-spatial connections.

### 2.3. Attention Mechanisms

An attention mechanism in image processing can pay weighted attention to different parts of the input data and involves assigning high weights to the main features in the input data. Many methods based on improved attention mechanism can pay multi-dimensional attention to the characteristics of the target sample. The self-attention mechanism introduced by the Transformer model is widely used in natural language processing tasks [36,37]. It assigns weighted attention to each position in the sequence by calculating the correlation between different positions in the input sequence. It enables the model to effectively capture long-range dependencies, leading to substantial performance improvements in tasks such as machine translation and text summarization.

Multi-head attention: The multi-head attention mechanism is an extension of the self-attention mechanism that computes different attentional representations in parallel by using multiple attention heads. Each attention head can focus on different aspects of the input sequence, thus improving the model’s ability to represent the input information. In this study, the SI-Attention mechanism is introduced to enhance the relevance of the multi-head subspace. Two attention mechanisms, channel and spatial, are also introduced in the DSFB module in the FeedForward neural network of this study [38,39]. The channel attention mechanism learns the importance of each channel for the features in each layer and strengthens or weakens the importance of different channels [40,41]. The spatial attention mechanism focuses on the correlation between different locations on the PRPD graph of each of the layers and weighs the importance of the different locations to obtain the weighted features of each layer. The attention mechanism can improve feature learning efficiency and model accuracy.

## 3. Proposed Method

For this topic, we need to categorize different localized discharge signals through the PMSNet structure to judge the four categories of the PRPD localized discharge signal maps. The neural network is trained on the PRPD maps, and the training parameters of the validation dataset are used to provide feedback on the training effect of PMSNet.

### 3.1. Overview of PMSNet

**Figure 3:**
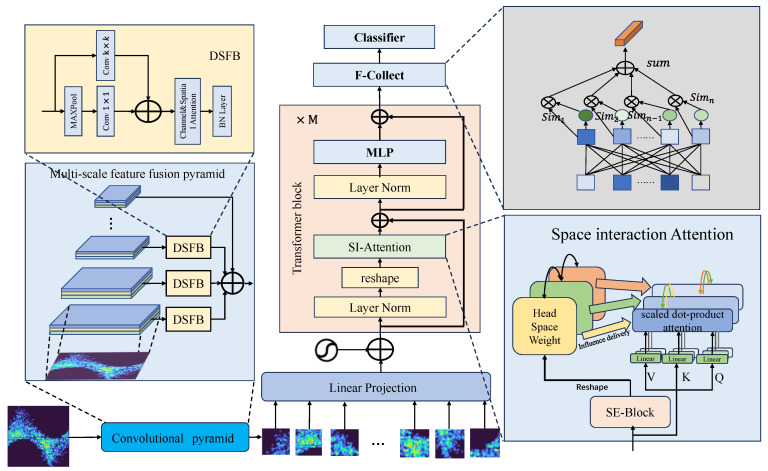
PMSNet structure.

The network structure of PMSNet is shown in Figure 3. The structure consists of three parts, namely, the multiscale feature fusion pyramid in the backbone network, the encoder module containing the SI-Attention module, and the F-Collect feature generation module. The input PRPD PD phase map is feature-extracted and feature-optimized by PMSNet.

The multiscale feature fusion pyramid extracts localized signal features at different scales for feature fusion. It is a kind of pyramid structure composed of a CNN and a DSFB module. The CNN gradually extracts the deep information, and the DSFB module extracts and strengthens the features of different fine-grained convolutional layers individually and sums the extracted features in the last layer of the convolutional pyramid to obtain fused features with different scales. SI-Attention strengthens the learning of the feature tensor to improve the interactions of the subspaces so that the features can sufficiently learn global and local information. The F-Collect module evaluates each feature vector by using the similarity-to-importance score, gives a corresponding weight to the vector by evaluation, and generates a classification vector for PD recognition.

#### 3.1.1. Multiscale Feature Fusion Pyramid

The multiscale feature fusion pyramid is the front-stem network of PMSNet and is composed of multiple convolutional layers, pooling layers, activation functions, and DSFB modules. The pyramid structure extracts shallow information through shallow convolution and then extracts deep features through continuous convolution, pooling, activation, and other operations. As the sensory field of the pyramid network continues to expand, it gradually begins to extract more overall abstract information, which tends to have a stronger ability to express information. For the classification task proposed in this study, only the output features of the last layer of the pyramid network are utilized for subsequent processing. The shallow features extracted by the pyramid structure can provide abundant high-resolution local detail information (e.g., vertical edge features, and horizontal edge features), and the use of these detail features can help the network to capture the subtle differences between classes. A multiscale feature fusion pyramid structure is therefore proposed, and the fusion features are obtained by optimizing and summing the features extracted by the pyramid at different scales through the multi-layer pyramid.

**Figure 4:**
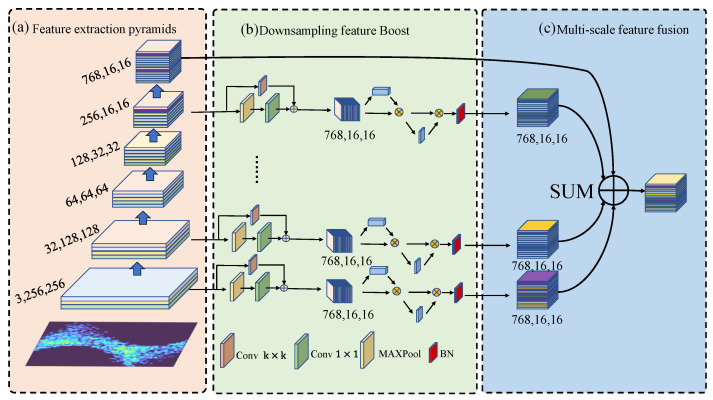
Feature pyramid network architecture: (**a**) feature fusion pyramid, (**b**) down-sampling feature boost module; and (**c**) multiscale feature fusion.

The multiscale feature fusion pyramid structure is shown in Figure 4a. The layers of the pyramid have  n layers, and each layer feature is denoted as $F_{i}$. The shallow features are denoted as $F_{1}$. In $F_{i}$, the size is reduced by the DSFB module to enhance the optimization of the information features of the current layer. A mixture of features is obtained by summing at the last layer.

#### 3.1.2. Down-Sampling Feature Boost Module

The DSFB module consists of two branches. The main branch is a maximum pooling layer to reduce the feature scale, and the other branch is a convolutional layer to expand the feature channel dimension $\left(1\times 1\right).$ The main branch is the largest pooling layer to reduce the feature scale, and the convolutional layer expands the channel dimension of the features. To avoid the information loss generated by the pooling operation when the features are down-sampled, a one-layer convolutional side branch is introduced next to the main branch inspired by the hopping connection, in which the convolutional layer’s convolutional kernel size is similar to that of the pooling layer’s convolutional kernel size in the main branch. The number of convolutional kernels in the convolutional layer is similar to that of the pooling layer in the main branch, which ensures that the dimensionality of the output features is similar to that of the main branch. The specific calculation process of down-sampling is as follows:

(1) $$F_{i}^{\prime }=MAX\left({Conv}_{1\times 1}\left(F_{i}\right)\right)+{Conv}_{k\times k}\left(F_{i}\right),\;\;\;\;\;\;\;\;\;i\in \left\{1,2,3,\ldots \ldots ,n\right\},\;i\neq \left(n-1\right)\&\left(n\right),\;\;$$

where $F_{i}$ is the characteristics of each layer of the pyramid network structure, ${Conv}_{1\times 1}$ represents the features of each layer of the 1×1 convolutional layer of the pyramid network structure, $C_{k\times k}$ refers to the features of each layer of the k×k convolutional layer, MAX denotes maximum pooling, and $F_{i}^{\prime }$ refers to the new feature map after down-sampling.

DSFB ensures that the $F_{i}(i\in \{1,2,3\ldots .n\})$ feature maps have the same size in scale and dimension as the last layer of the network’s ($F_{n}$). At the same time, it minimizes the loss of some information during down-sampling. After down-sampling, a small, shallow feature tensor is obtained, and to compensate for the feature loss caused by the reduced size, we enhance the shallow feature representation by adding channel and spatial attention mechanisms after each down-sampling module. As shown in Figure 4b, this enhancement involves two main steps, namely, channel and spatial attention. The channel attention mechanism enhances or suppresses the channel features on the basis of the importance of the feature channel, and the spatial attention mechanism focuses on the importance of the feature in the spatial location.

(a) Channel attention mechanism: The feature map is $F_{i}^{\prime }$. Global average pooling is performed, and the feature map of each channel is compressed into a scalar value, which is processed through the fully connected layer and activation function to obtain the channel attention weights. The channel attention weight scales the features on each channel to enhance the feature representation. The input feature maps are $F_{i}^{\prime }$. Global average pooling and global maximum pooling are performed to obtain two 1×1×C feature maps. The two feature maps are fed into a two-layer fully connected neural network for summation, and the sigmoid function is used to obtain the weight coefficients between the two layers. These coefficients are subsequently multiplied with the input of $F_{i}^{\prime }$. This multiplication process is specifically expressed as follows:

(2) $$M_{c}\left(F_{i}^{\prime }\right)=Sigmoid\left(MLP\left(AugPool\left(F_{i}^{\prime }\right)\right)+MLP\left(MaxPool\left(F_{i}^{\prime }\right)\right)\right)$$

The weight coefficients of the attention are determined by the channel attention mechanism along the channel dimensions, weighted with the original features, and expressed as:

(3) $$F_{i}^{\prime \prime }=F_{i}^{\prime }*M_{c}\left(F_{i}^{\prime }\right),\;\;\;\;i\in \left\{1,2,3,\ldots ,n\right\}\;.\;$$

(b) Spatial attention mechanism: The spatial attention mechanism is included in the convolutional attention mechanism and used to adjust the importance of feature representations at different spatial locations in the feature. The inputs are the channel attention weights with $F_{i}^{\prime \prime }\in R^{H\times W\times C}$. Maximum pooling and average pooling of one channel dimension are performed to derive two H×W×1 feature maps. The two feature maps are spliced to obtain H×W×2, which are reduced to one channel by one convolutional layer by using the 7×7 convolution kernel. H and W are kept constant, and the output is H×W×1. The size of the feature map is determined, and the sigmoid function is used to generate the spatial weight coefficients, which are then multiplied with the input feature map to obtain the final feature map containing channel and spatial attention. The expression is as follows:

(4) $$M_{s}\left(F_{i}^{\prime \prime }\right)=Sigmoid\left({Conv}_{7\times 7}[AugPool\left(F_{i}^{\prime \prime }\right);MaxPool\left(F_{i}^{\prime \prime }\right)])\right),$$

where ${Conv}_{7\times 7}$ denotes 7 × 7 convolution. The new features are obtained through the whole convolutional attention module and expressed as follows:

(5) $$F_{i}^{\prime \prime \prime }=F_{i}^{\prime \prime }*M_{s}\left(F_{i}^{\prime \prime }\right),\;\;\;\;i\in \left\{1,2,3,\ldots ,n\right\}\;,\;$$

where $F_{i}^{\prime \prime \prime }$ is the global feature that represents the reduced size of each level, and it encompasses spatial and channel attention at the corresponding scale.

The final feature map $F_{n}$ with the corresponding elements of the middle- and low-dimensional feature maps that have passed through the down-sampling module is employed to achieve multiscale feature fusion. The multiscale feature fusion pyramid calculation formula is shown as follows:

(6) $$F_{s}=F_{1}^{\prime \prime \prime }+F_{2}^{\prime \prime \prime }+F_{3}^{\prime \prime \prime }+\cdots +F_{n},\;$$

where $F_{s}$ represents the final multiscale fused features. The topmost and penultimate layers of the feature pyramid are not down-sampled.

#### 3.1.3. Spatial Interaction Multiple Attention Module

**Figure 5:**
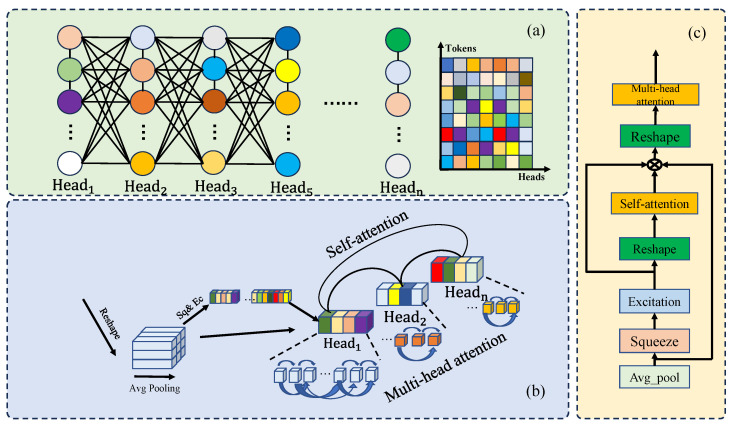
Structure of the spatial attention mechanism: (**a**) weighting chart, (**b**) structure of the SI-Attention mechanism; and (**c**) SI-Attention flowchart.

Not all the information in the input localized signal sequence vectors exerts a considerable effect on the classification task. The attention mechanism can assign a large weight to finding key information from a large number of pieces of information in the Transformer model by using multi-head self-attention in feature vector interactive learning. Through the linear mapping of the input, the original feature space is decomposed into several subspaces, each of which is a head. Each head learns and attends to different local features of the input sequence separately to extract rich information. Then, the final multi-head attention representation is obtained by splicing or weighted averaging the outputs of these heads. In this study, SI-Attention is proposed, which can spatially establish head-to-head relationships and enhance the feature representation of each subspace globally. The specific structure of SI-Attention is shown in Figure 5.

The vectors are inputted to the decoder to reconstitute (B,C,H,W) of the feature tensor, where B is the batch size and C is the number of channels. In accordance with the tensor size, global average pooling of the channels is performed by changing H×W to 1×1 of the channels to obtain the tensor size (B,C,1,1). The corresponding size in this study is (B,768,1,1), and it is specifically expressed as:

(7) $$a={Rp}_{\left(B,C\right)}\left(AVG_{POOL\left(x\right)}\right),\;$$

where ${Rp}_{\left(B,C\right)}$ represents the tensor decomposition, $\left(B,C\right)$ is the size dimension of the tensor, and AVG_POOL represents global adaptive pooling. The two compressed and extended FC layers learn channel C of attention represented as:

(8) $$H=FC\left(a\right),$$

where FC represents the compression and reduction operations performed on channel *C* that yields a dimension of (B,C) of the H tensor. The *H* tensor represents the spatial feature weight distribution and the spatial upper relation of the input vectors. In accordance with the number of multi-head attention subspaces (number of heads), the H tensor is segmented, and the number of segments corresponds to the number of heads to obtain a new dimension size of (B,Heads,C/Heads) of the vector. Self-attention is applied to the new tensor to find the correlation between subspaces. The self-attention of the features of each representative subspace is calculated to derive the weight representation of the subspace relative to the global space, and the calculated weights with the elements in the corresponding segments are presented as:

(9) $$\left\{\begin{array}{c} {head}_{i}=Rp\left(B,Heads,\frac{c}{Heads}\right)\\ W_{i}=Self-Attention\left(\left[{head}_{1},{head}_{2},\ldots ,{head}_{n}\right]\right), \end{array}\right.$$

where ${head}_{i}$ is the spatial feature vector representing each head and $W_{i}$ is the spatial weight of the corresponding head. By combining weights $W_{i}\times H_{i}$, the interaction between spaces is enhanced by reconstructing the vectors endowed with weights as the (B,C,H∗W) feature tensor. The feature vector (B,C,H∗W) is mapped to the i header subspaces and then mapped by three matrices (query, key-value, and value matrices) into $W_{i}H_{i}$ in the subspace of the header. After multi-head attention, the result is outputted after cross-attention. These procedures are defined as:

(10) $$\left\{\begin{array}{c} WH=\left(\left[q_{1},\ldots ,q_{n}\right],\left[k_{1},\ldots ,k_{n}\right],\left[v_{1},\ldots ,v_{n}\right]\right)\\ W_{i}H_{i}=softmax\left(\frac{Q_{i}K_{i}^{T}}{\sqrt{d}}\right)V_{i}\\ output=MHA\left(W_{1}H_{1},\ldots ,W_{i}H_{i}\right), \end{array}\;\right.$$

where WH represents the head space and MHA is the multi-head attention mechanism.

The spatial multi-head attention mechanism saves time and reduces memory utilization by matching weights to each head on the basis of the number of heads for parallel computation. The spatial attention architecture concatenates the weights within the subspace and the weights between the heads to optimize the learning effect of the feature vectors by double-weighting.

#### 3.1.4. Feature Aggregation Module

**Figure 6:**
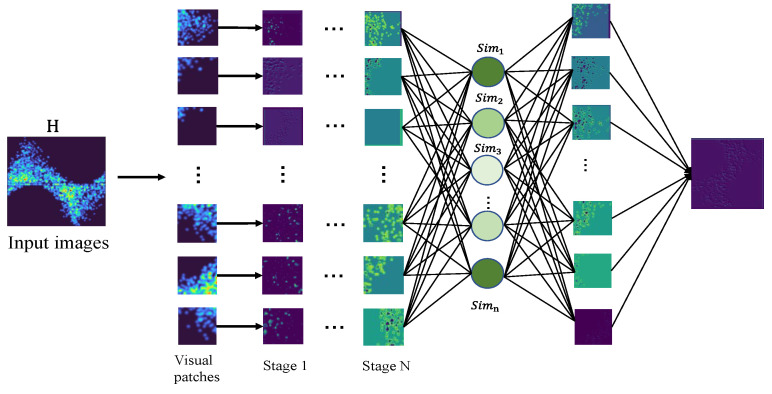
Structure of the feature aggregation module.

This network structure selects a classification token. In the Vision Transformer model, the authors add a flag-like cls_token to follow the rest of the tokens to learn the training features before inputting them to the encoder and finally classify using the class token. We perform the classification prediction task by using the original token without cls_token. The most common method is the average way because the relationship between the query and key in the attention mechanism can be adjusted by adaptively adjusting the weight of the feature aggregation. The tie-breaking way assigns the same weight to all key values, which limits the expressive ability of the model. The original token is based on a feature of the original map and continues to learn the global features, from which a feature vector is selected, or a vector is obtained by setting the threshold. The conditions for feature selection cannot fully express all the features of the global token; therefore, this study proposes a feature generation module that considers the global feature token through the similarity between the features in order to give all the vectors an importance score. The higher the similarity of a single feature vector is to the global one, the higher the proportion of features is in the global one, and the higher the importance score is. On the contrary, a vector with low similarity proves that the features it contains still cannot obtain a large weight after the attention mechanism. This kind of feature volume has a high proportion of useless features, so we give it a low score. By transforming the importance score to perform feature aggregation, we derive a reasonable classification feature vector. The structure of the feature generation module, F-Collect, is shown in Figure 6.

The correlation and similarity of the original features are utilized to mine the essence of the token, obtain all the features inside the most representative global feature vector, and give all the tokens a score. A high score proves that the features are significantly densely populated. When the weight is large, the calculation of the similarity between the 1D vectors uses cosine similarity (Cosine). The calculation of similarity is shown in Equation (11) as follows:

(11) $$S\left(m,n\right)=\frac{m\cdot n}{\left\|m\right\|\cdot \left\|n\right\|}=\frac{\sum_{i=1}^{d}m_{i}\cdot n_{i}}{\sqrt{\sum_{i=1}^{d}m_{i}}\cdot \sqrt{\sum_{i=1}^{d}n_{i}},}$$

where $m\in R^{d}$ and $n\in R^{d}$ belong to two vectors inside tokens.$\;S\left(m,n\right)$ denotes the similarity of the two vectors. The formula for calculating the similarity between the two can be expressed as:

(12) $${Sim}_{cos}\left(q_{i},q_{j}\right)=S_{c}\left(q_{i},q_{j}\right),\;\;\;i,j\in \left[1,2,3,\ldots ,n\right]\;.\;\;$$

The similarity matrix can be obtained from the similarity value between two vectors and expressed as:

(13) $$S_{n\times n}=\left[\begin{array}{ccc} 1 & \ldots & Sim\left(q_{i},q_{n}\right)\\ \vdots & \ddots & \vdots \\ Sim\left(q_{i},q_{1}\right) & Sim\left(q_{i},q_{j}\right) & Sim\left(q_{i},q_{n}\right)\\ Sim\left(q_{n},q_{1}\right) & \ldots & 1 \end{array}\right],$$

where $Sim\left(q_{i},q_{j}\right)$ is the similarity computation between the ith vector and the jth vector and $S_{n\times n}$ represents the similarity matrix. The elements in the similarity matrix are summed column by column along one dimension to obtain each feature representation as follows:

(14) $$Sum\left(S\;,dim=1\right)=\left[\begin{array}{c} \begin{array}{c} \sum_{j=1}^{n}S_{1j}\\ \sum_{j=1}^{n}S_{2j}\\ \vdots \end{array}\\ \sum_{j=1}^{n}S_{nj} \end{array}\right],\;$$

where dim=1 denotes the first dimension of the matrix and  SumS denotes the result obtained by summing the elements of each row. The Softmax function is employed to convert the similarity of each feature vector into an importance score, and the process can be expressed as:

(15) $$Softmax\left(S_{i}\right)=\frac{\exp\left(S_{i}\right)}{\sum_{j}\exp\left(S_{j}\right),}$$

(16) $${Scores}_{i}=Softmax\left(Sum\right),$$

The vector is multiplied with the corresponding element in the matrix and summed to obtain the optimized classification feature vector. The specific expression is as follows:

(17) $$G\_Token=\sum_{i=1}^{n}T_{i}{Scores}_{i},\;$$

where G_token is the optimized classification feature and $T_{i}$ is each feature vector.

## 4. Experimental Results and Discussion

### 4.1. Partial-Discharge Dataset

The dataset for this work contains PD data collected by sensors. The dataset used in the experiment is the PRPD map of PDs, which is composed of the number of PDs, phase, and discharge amplitude. It contains four types of discharges: surface, internal, metal particle, and suspended discharges. The experiment investigates the recognition of the four discharge types by building a network structure and then tests the robustness and recognition accuracy of the network by comparing it with other recognition networks. The dataset images have a uniform input size of 256×256×3. The dataset has a total of 11,000 PRPD images. The training, validation, and test datasets have a ratio of 6:3:1.

### 4.2. Comparative Identification Experiments

PMSNet is an architecture network of Transformer that utilizes a combination of CNN convolution and pooling layers to effectively capture different scales and levels of features in the image and extract representative features. In this study, we individually perform down-sampling feature enhancement processing (DSFB) on each layer of scaled features to obtain multiscale fusion features through the fusion of different fine-grained features. Spatial weights are assigned to the features by SI-Attention in Transformer. The classification features are generated through the feature generation module, and the results are outputted.

To further demonstrate the performance of PMSNet in the recognition of PD signals, we compared it with other methods. We placed mainstream neural networks in our PRPD dataset for comparative experiments. The comparison results are shown in Table 1. We compared the following recognition methods:

BPNN [42]: It is an applied method for identifying types of PDs by using artificial neural networks (ANNs) The authors utilize this branch of ANN for improved diagnosis of high-voltage devices, which is performed by observing the phase pattern of PD signals. The PD signals are evaluated by observing the maximum amplitude of positive and negative PDs and the number of times PDs appear in each cycle. An accuracy of 73.3% is achieved in the recognition task;

BPNN (PCA) [43]: Khan et al. proposed a technique that combines backpropagation artificial neural network (BP-ANN) with principal component analysis (PCA) to enhance localized discharge pattern recognition. The results demonstrate a considerable improvement in the performance of BP-ANN through the integration of PCA. Without PCA, the accuracy of BP-ANN is 78% and 80% for one and two hidden layers, respectively. However, by incorporating PCA, both cases achieve reduced execution time while maintaining a fairly high accuracy of 78.1%;

FL [44]: Zeng et al. proposed a method to assess the importance of PD by using the statistical features of two-level FIS and PRPD data. High accuracy was achieved. Duan et al. proposed a PD identification method for XPLE cables on the basis of parameter optimization of a support vector machine (SVM), which uses fractal features extracted from PRPD as the input;

GA-SVM [45]: A genetic algorithm is used to optimize the parameters of SVM. The diagnostic accuracy is considerably improved compared with that of SVM before optimization. Effective processing of small-sample PD data through the powerful flourishing capability of GA-SVM produces a recognition accuracy of up to 80%;

CNN-LSTM [46]: The input for identifying PDs consists of a dual-channel image jointly constructed with PRPD and PRPS signals. This novel approach utilizes the dual-channel spectrum of discharge signals as a joint driver to optimize the neural network for feature extraction, employing a hybrid deep learning model of CNN and LSTM. As a result, our method achieves remarkable efficiency in the identification task;

DCGAN-YOLOv5 [47]: It is a multisource PD detection algorithm based on deep convolutional adversarial network and YOLOv5. It incorporates spatial and channel attention mechanisms in the feature extraction network to improve recognition accuracy and efficiency. When spatial and channel attention mechanisms are added to the network, the recognition accuracy reaches 80.2% and 83.3% in single- and multi-source PD signals, respectively;

CBAM-Resnet [48]: The convolutional attention residual network for GIS PD pattern recognition (CBAM-Resnet) that uses PRPD images as an input to the model is employed to obtain four classification features.

In this study, the PRPD dataset is used to conduct comparative experiments on VGG, VIT, Swim-Transformer, mainstream CNN architectures, and VIT structures. The comparison results are shown in Table 2.

**Figure 7:**
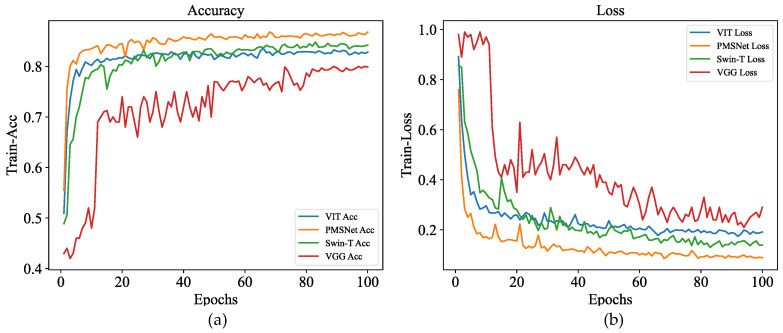
Comparison of the accuracy in different epochs: (**a**) accuracy of the four models; and (**b**) loss values of the four models.

We conducted a comparative experiment on the four kinds of networks in Table 2, with the results showing that PMSNet’s accuracy is the highest. The four networks’ accuracy in 100 epochs and the loss value changes are shown in Figure 7.

#### 4.2.1. Classification Validation

**Figure 8:**
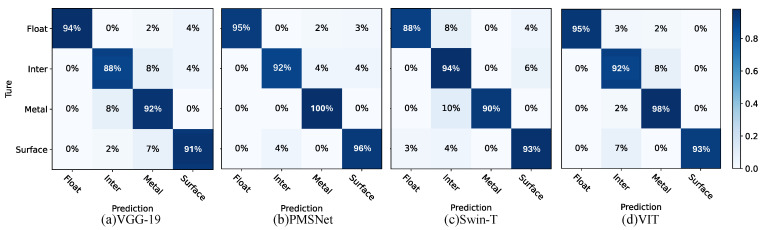
Confusion matrix comparison: (**a**) VGG-19; (**b**) PMSNet; (**c**) Swin-T; and (**d**) VIT.

According to the image, PMSNet has the highest accuracy after 100 epochs and the lowest training loss value among the four models. A recognition test was then performed, and a confusion matrix was obtained as the result (Figure 8). The comparative experiments indicate that PMSNet achieves relatively good results in the PD task, as shown in Figure 8b, and it has the best prediction accuracy in the four PD categories.

#### 4.2.2. Visualization

**Figure 9:**
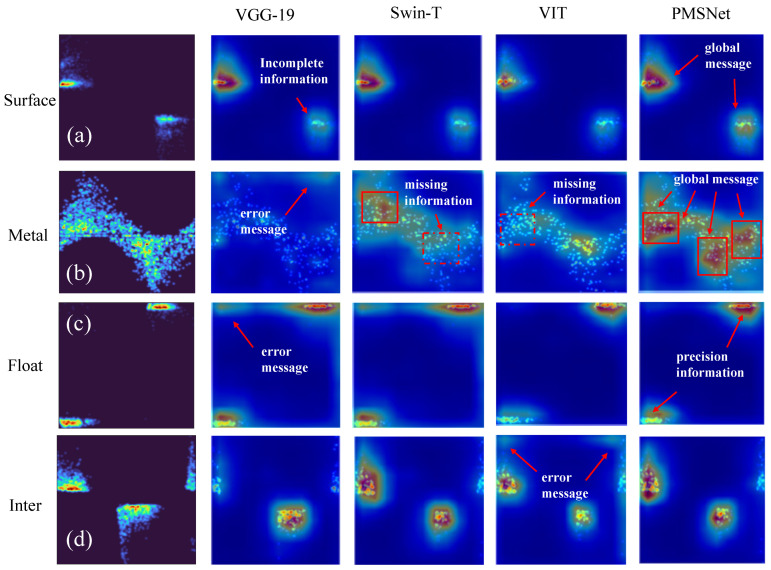
Multiple network visualizations.

In order to reflect the good performance of the PMSNet network and observe the difference between this network and other networks in focusing on local areas, this paper uses a large number of visualization experiments to prove the interpretability of PMSNet. Figure 9 shows some examples of ScoreCAM that clearly indicate the regions of interest for different inputs. We selected four different classes of PRPD images for classification and four methods (i.e., VGG-19, Swin-T, VIT, and PMSNet) for comparison. In Figure 9, the red arrows indicate additional interpretations, the invariant cues identified by PMSNet in PRPD images are marked by red solid boxes, and missing important information is marked by dashed boxes. In the visualization, the higher the network pays attention to the region in the sample data, the darker the color is. The corresponding feature should be an important feature, if not, it represents the insufficient understanding ability of the model. As can be seen from the first three columns in Figure 9, our method better recognizes global core features compared with general CNN- or VIT-based methods. In detail, our method encompasses global features and accurately recognizes local features, which other networks cannot do. As shown in Figure 9b, VGG-19 is unable to localize the features, and the model’s generalization ability is insufficient, especially in the case where the recognition of the remaining kinds of PRPD discharges is incorrect or incomplete. VIT and Swin-T focus on local information, as shown in Figure 9a,b, and they incorrectly adopt the local features as the representative recognition features, leading to bias. By contrast, our method successfully connects the local and global features. In Figure 9a, we accurately identify two unique local features, which are precisely the unique feature distributions of the surface discharge signal. In Figure 9b, the SI-Attention mechanism plays a crucial role, and the identification features focus on the global discharge number of intensive places and identify them successfully. These results indicate that our method can learn the long-dependency semantic relations of complex PRPD graphs. Figure 9c,d shows that our method can recognize the core features of different PRPD graphs.

#### 4.2.3. Differences in the Effects of Various Model Parameters

Ablation experiments were performed on the number of pyramid layers of the feature fusion pyramid. The more layers of the pyramid there are, the slower the change is from shallow to deep features. When many features on different levels are fused, the dimension of the input PRPD map is 3×256×256 (bottom of the pyramid), and the tensor dimension of the last layer is 768×16×16. The larger the number of pyramid layers is, the larger the number of DSFB modules that are pulled out per layer and the more final fused multiscale features there are. The results are shown in Table 3.

The results indicate that when the number of layers of a multiscale feature fusion pyramid tower is varied, training accuracy is affected. The general accuracy slightly decreases when the same number of layers of the pyramid structure is used, when the down-sampling module is added, and when the accuracy of the difference is not added. The first four layers of the five-layer pyramid are individually processed with the DSFB module separately, and the best results are achieved by performing feature fusion. From the results, we find that the five-layer pyramid structure fuses multiscale features compared with the four-layer pyramid structure that fuses detailed features, which indirectly affects the training effect. However, increasing the number of layers does not produce the best training effect; when the number of layers increases to six, the training accuracy does not increase. Analysis of the training to the last few layers indicates that the deep features contain the main features, and the features at the scale it belongs to account for a low percentage; these features overlap with a large number of features in the last layer of the pyramid, affecting the subsequent results.

By adding a learnable variable to make a cls_token, and by using the F-Collect module to generate a categorized token, we performed a comparison in 50 and 100 epochs, respectively. The results are shown in Table 4.

**Figure 10:**
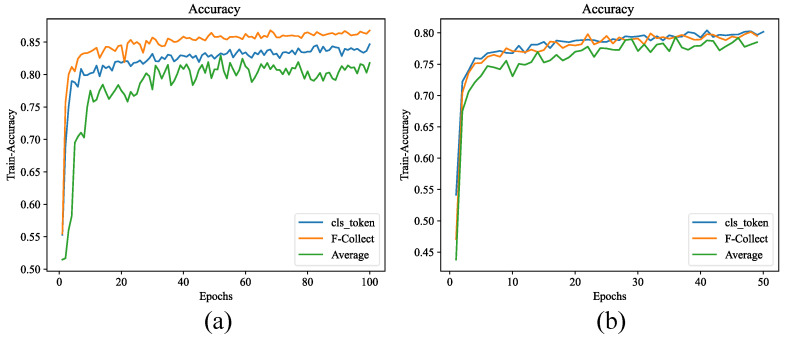
Comparison of training accuracy in: (**a**) 100 epochs; and (**b**) 50 epochs.

The comparison experiment in Figure 10 shows that no difference exists between the two models in 50 epochs, but the accuracy of the model that uses the F-Collect module in 100 epochs is 3–4% higher than that of the model that uses the cls_token module. The model reaches convergence in 100 epochs, and the advantage of F-Collect is highlighted. The learning rate for this experiment is set to $1\times 10^{-3}$; when a larger learning rate is used, the training becomes unstable and cannot converge. When we decrease the learning rate, the training time increases considerably, causing the model training to be stuck in a certain solution and unable to learn the optimal parameter combination.

**Figure 11:**
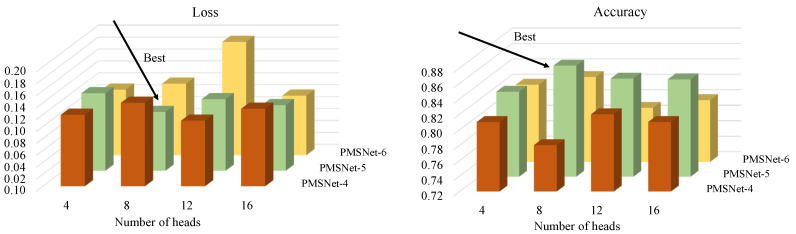
Performance of SI-Attention with different number of heads in PMSNet.

The SI-Attention attention mechanism proposed in this study is set up with a subspace of eight heads. The more heads there are, the greater the number of corresponding spatial interactions. We conducted ablation experiments to compare the networks PMSNet-4, PMSNet-5, and PMSNet-6 with different pyramid layers under 4, 8, 12, and 16 heads, and the results are shown in Figure 11. PMSNet-4, PMSNet-5, and PMSNet-6 represent the 4th, 5th, and 6th layers of the feature pyramid in the front-stem network. According to Figure 11, PMSNet-5 performs optimally when the number of heads in SI-Attention is 8.

## 5. Conclusions

This study investigates in detail how to introduce convolution into the Vision Transformer architecture and implement multiscale feature presentation through convolution to combine the advantages of Transformer with those of CNN for image recognition tasks. The multiscale fusion pyramid structure was built by convolution, and each layer of the pyramid was individually feature-extracted and fused by the down-sampling module to obtain the fused features. The spatial multi-attention mechanism was applied to the fused features to enhance the spatial interactive learning among the features, in addition to the final generation of classification features by the feature aggregation module. The experiments show that the multiscale feature fusion pyramid network has the highest recognition accuracy and efficiency with the PRPD dataset among the compared networks. The method is advanced and innovative with respect to partial-discharge signal recognition and has achieved some success in this field. It is also the first innovation and application of the network jointly constructed by CNN and Transformer in the field of partial-discharge signal recognition. In the future, we will focus on localized discharge signal recognition network lightweighting and multimodal learning to deploy the network on devices.

## Figures and Tables

**Table 1:** Comparison of the recognition accuracy of PMSNet and that of advanced PRPD recognition methods.

Method	Data Type	Acc (%)
BPNN [42]	PRPD	73.3
BPNN(PCA) [43]	PRPD	78.1
FL [44]	PRPD	79.7
GA-SVM [45]	PRPD	80.2
CNN-LSTM [46]	PRPD + PRPS	78.7
DCGAN-YOLOv5 [47]	PRPD	83.2
CBAM-Resnet [48]	PRPD	81.3
PMSNet (ours)	PRPD	**85.2**

**Table 2:** Comparison of the accuracy of PMSNet with that of typical high-performance networks.

Method	Input Size	Acc (%)
VIT [33]	256×256	81.1
Swim-T [49]	256×256	82.7
VGG-19 [50]	256×256	79.8
PMSNet (ours)	256×256	**85.2**

**Table 3:** Different accuracy rates corresponding to pyramid layers.

Pyramid Scheme	Number of DSFBs	Accuracy (%)
4	0	80.2
4	3	82.2
5	0	81.8
5	4	**85.1**
6	0	82.8
6	5	81.3

**Table 4:** Compare the accuracy of using cls-token, F-Collect and average method.

Epochs	cls_token	F-Collect	Average	Accuracy (%)
50	✓	×	×	79.8
50	×	✓	×	79.3
50	×	×	✓	78.1
100	✓	×	×	81.0
100	×	✓	×	**85.4**
100	×	×	✓	80.2

## Data Availability

Restrictions apply to the datasets.

## References

[1] Hussain G.A., Hassan W., Mahmood F. (2023). Review on Partial Discharge Diagnostic Techniques for High Voltage Equipment in Power Systems. IEEE Access.

[2] Acheen R. Risk assessment related to PD activity in electrical insulation systems of machines in flammable environment. Proceedings of the AIAA AVIATION 2023 Forum.

[3] Bandara S., Rajeev P., Gad E. (2023). Power Distribution System Faults and Wildfires: Mechanisms and Prevention. Forests.

[4] Stone G.C. (2012). A perspective on online partial discharge monitoring for assessment of the condition of rotating machine stator winding insulation. IEEE Electr. Insul. Mag..

[5] Carvalho I.F., da Costa E.G., Nobrega L.A.M.M., Silva A.D.d.C. (2024). Identification of Partial Discharge Sources by Feature Extraction from a Signal Conditioning System. Sensors.

[6] Beura C.P., Wolters J., Tenbohlen S. (2024). Application of Pathfinding Algorithms in Partial Discharge Localization in Power Transformers. Sensors.

[7] Riera-Guasp M., Antonino-Daviu J.A., Capolino G.A. (2015). Advances in electrical machine, power electronic, and drive condition monitoring and fault detection: State of the art. IEEE Trans. Ind. Electron..

[8] Choudhary M., Shafiq M., Kiitam I., Palu I., Hassan W., Singh P.P. (2024). Investigation of partial discharge characteristics in XLPE cable insulation under increasing electrical stress. Eng. Fail. Anal..

[9] Monzón-Verona J M., González-Domínguez P., García-Alonso S. (2024). Characterization of Partial Discharges in Dielectric Oils Using High-Resolution CMOS Image Sensor and Convolutional Neural Networks. Sensors.

[10] Deng Y., Zhu K., Zhao G. (2022). Efficient partial discharge signal denoising method via adaptive variational modal decomposition for infrared detectors. Infrared Phys. Technol..

[11] Okubo H., Hayakawa N. (2005). A novel technique for partial discharge and breakdown investigation based on current pulse waveform analysis. IEEE Trans. Dielectr. Electr. Insul..

[12] Hoshino T., Koyama H., Maruyama S., Hanai M. (2006). Comparison of sensitivity between UHF method and IEC 60270 for onsite calibration in various GIS. IEEE Trans. Power Deliv..

[13] Tenbohlen S., Denissov D., Hoek S., Markalous S.M. (2008). Partial discharge measurement in the ultrahigh frequency (UHF) range. IEEE Trans. Dielectr. Electr. Insul..

[14] Gao W., Ding D., Liu W. (2011). Research on the typical partial discharge using the UHF detection method for GIS. IEEE Trans. Power Deliv..

[15] Fang W., Chen G., Li W., Xu M., Xie W., Chen C., Wang W., Zhu Y. (2023). A PRPD-Based UHF Filtering and Noise Reduction Algorithm for GIS Partial Discharge. Sensors.

[16] Albarracín-Sánchez R., Álvarez-Gómez F., Vera-Romero C.A. (2020). Separation of partial discharge sources measured in the high-frequency range with HFCT sensors using PRPD-teff patterns. Sensors.

[17] Liu H., Fang S., Zhang Z., Li D., Lin K., Wang J. (2022). MFDNet: Collaborative Poses Perception and Matrix Fisher Distribution for Head Pose Estimation. IEEE Trans. Multimed..

[18] Satish L., Zeng l. (1994). Artificial neural networks for recognition of 3-d partial discharge patterns. IEEE Trans. Dielectr. Electr. Insul..

[19] Strachan S.M., Rudd S., McArthur S.D., Judd M.D., Meijer S., Gulski E. (2008). Knowledge-based diagnosis of partial discharges in power transformers. IEEE Trans. Dielectr. Electr. Insul..

[20] Li J., Liao R., Grzybowski S., Yang L. (2010). Oil-paper aging evaluation by fuzzy clustering and factor analysis to statistical parameters of partial discharges. IEEE Trans. Dielectr. Electr. Insul..

[21] Liu H., Zheng C., Li D., Zhang Z., Lin K., Shen X., Xiong N.N., Wang J. (2022). Multi-perspective social recommendation method with graph representation learning. Neurocomputing.

[22] Zhang Q., Lin J., Song H., Sheng G. (2018). Fault Identification Based on PD Ultrasonic Signal Using RNN, DNN and CNN. 2018 Condition Monitoring and Diagnosis (CMD).

[23] Zhang A., He J., Lin Y., Li Q., Yang W., Qu G. (2020). Recognition of partial discharge of cable accessories based on convolutional neural network with small data set. COMPEL-Int. J. Comput. Math. Electr. Electron. Eng..

[24] Kumar H., Shafiq M., Kauhaniemi K., Elmusrati M. (2024). A Review on the Classification of Partial Discharges in Medium-Voltage Cables: Detection, Feature Extraction, Artificial Intelligence-Based Classification, and Optimization Techniques. Energies.

[25] Li J., Jiang T., Harrison R., Grzybowski S. (2012). Recognition of ultra high frequency partial discharge signals using multi-scale features. IEEE Trans. Dielectr. Electr. Insul..

[26] Sukma T.R., Khayam U., Sugawara R. (2018). Classification of partial discharge sources using waveform parameters and phase-resolved partial discharge pattern as input for the artificial neural network. Proceedings of the 2018 Condition Monitoring and Diagnosis (CMD).

[27] Herath Comparison of supervised machine learning techniques for PD classification in generator insulation. Proceedings of the 2017 IEEE International Conference on Industrial and Information Systems (ICIIS).

[28] Michał K., Daria W. (2019). A Classification Method for Select Defects in Power Transformers Based on the Acoustic Signals. Sensors.

[29] Li G., Wang X., Li X., Yang A., Rong M. (2018). Partial Discharge Recognition with a Multi-Resolution Convolutional Neural Network. Sensors.

[30] Puspitasari N., Khayam U., Suwarno, Kakimoto Y., Yoshikawa H., Kozako M., Hikita M. (2019). Partial discharge waveform identification using image with convolutional neural network. Proceedings of the 2019 54th International Universities Power Engineering Conference (UPEC).

[31] Liu H., Zhang C., Deng Y., Xie B., Liu T., Zhang Z., Li Y. (2023). TransIFC: Invariant Cues-aware Feature Concentration Learning for Efficient Fine-grained Bird Image Classification. IEEE Trans. Multimed..

[32] Vaswani A., Shazeer N., Parmar N., Uszkoreit J., Jones L., Gomez A.N., Kaiser L., Polosukhin I. (2017). Attention is all you need. Adv. Neural Inf. Process. Syst..

[33] Dosovitskiy A., Beyer L., Kolesnikov A., Weissenborn D., Zhai X., Unterthiner T., Dehghani M., Minderer M., Heigold G., Gelly S. An Image is Worth 16x16 Words: Transformers for Image Recognition at Scale. Proceedings of the International Conference on Learning Representations.

[34] Yuan L., Chen Y., Wang T., Yu W., Shi Y., Jiang Z., Tay F.E.H., Feng J., Yan S. Tokens-to-token vit: Training vision transformers from scratch on imagenet. Proceedings of the IEEE/CVF International Conference on Computer Vision.

[35] Wu H., Xiao B., Codell N. Cvt: Introducing convolutions to vision transformers. Proceedings of the IEEE/CVF International Conference on Computer Vision.

[36] Liu H., Zhang C., Deng Y., Liu T., Zhang Z., Li Y. (2023). Orientation Cues-Aware Facial Relationship Representation for Head Pose Estimation via Transformer. IEEE Trans. Image Process.

[37] Liu H., Liu T., Chen Y., Zhang Z., Li Y. (2024). EHPE: Skeleton Cues-based Gaussian Coordinate Encoding for Efficient Human Pose Estimation. IEEE Trans. Multimedia.

[38] Woo S., Park J., Lee J.Y. Cbam: Convolutional block attention module. Proceedings of the European Conference on Computer Vision (ECCV).

[39] Wang Q., Wu B., Zhu P. ECA-Net: Efficient channel attention for deep convolutional neural networks. Proceedings of the IEEE/CVF Conference on Computer Vision and Pattern Recognition.

[40] Liu T., Liu H., Yang B., Zhang Z. (2024). LDCNet: Limb Direction Cues-aware Network for Flexible Human Pose Estimation in Industrial Behavioral Biometrics Systems. IEEE Trans. Ind. Inf..

[41] Zhang C., Liu H., Deng Y., Xie B., Li Y. TokenHPE: Learning Orientation Tokens for Efficient Head Pose Estimation via Transformers. Proceedings of the 2023 IEEE/CVF Conference on Computer Vision and Pattern Recognition (CVPR).

[42] Lunetta L.S., Khayam U., Maulana R. (2019). Design of pattern recognition application of partial discharge signals using artificial neural networks. Proceedings of the 2019 International Conference on Electrical Engineering and Informatics (ICEEI).

[43] Khan Y. (2016). Partial discharge pattern analysis using PCA and back-propagation artificial neural network for the estimation of size and position of metallic particle adhering to spacer in GIS. Electr. Eng..

[44] Zeng F., Dong Y., Tang J. (2015). Feature extraction and severity assessment of partial discharge under protrusion defect based on fuzzy comprehensive evaluation. IET Gener. Transm. Distrib..

[45] Duan Y., Zhang H., Hu X. (2019). PD pattern recognition of XLPE cable based on parameter optimal support vector machine algorithm. Proceedings of the 2019 14th IEEE Conference on Industrial Electronics and Applications (ICIEA).

[46] Zheng Q., Wang R., Tian X., Yu Z., Wang H., Elhanashi A., Saponara S. (2023). A real-time transformer discharge pattern recognition method based on CNN-LSTM driven by few-shot learning. Electr. Power Syst. Res..

[47] Wu M., Jiang W., Shen D., Luo Y., Yang J. (2022). Multi-ource partial discharge pattern recognition algorithm based on DCGAN-Yolov5. IEEE Trans. Power Deliv..

[48] Hu D., Chen Z., Yang W., Zhu T., Ke Y. Partial Discharge Pattern Recognition of GIS Based on CBAM-ResNet. Proceedings of the 2022 4th International Conference on Robotics, Intelligent Control and Artificial Intelligence.

[49] Liu Z., Lin Y., Cao Y. Swin transformer: Hierarchical vision transformer using shifted windows. Proceedings of the IEEE/CVF International Conference on Computer Vision.

[50] Simonyan K., Zisserman A. (2014). Very deep convolutional networks for large-scale image recognition. arXiv.

